# Prevalence and epidemiological correlates and treatment outcome of HCV infection in an Italian prison setting

**DOI:** 10.1186/1471-2458-13-981

**Published:** 2013-10-20

**Authors:** Micaela Brandolini, Stefano Novati, Annalisa De Silvestri, Carmine Tinelli, Savino Francesco Antonio Patruno, Roberto Ranieri, Elena Seminari

**Affiliations:** 1 Clinica di Malattie Infettive, Fondazione IRCCS Policlinico San Matteo, Viale Camillo Golgi 19, 27100 Pavia, Italy; 2 Servizio di Biometria e Statistica, Fondazione IRCCS Policlinico San Matteo, Viale Camillo Golgi 19, 27100 Pavia, Italy; 3 Divisione di Malattie Infettive e Tropicali, Fondazione IRCCS Policlinico San Matteo, Viale Camillo Golgi 19, 27100 Pavia, Italy; 4 Medicina Protetta Ospedale San Paolo-Milano, Milan, Italy

**Keywords:** HCV-infection, HCV-prevalence, Prison, HCV treatment, HIV-HCV coinfection

## Abstract

**Background:**

The aim of the present study is to test in the feasibility of a screening programme for HCV infection in an Italian prison and to evaluate the treatment outcomes.

**Method:**

Single-centre cross-sectional study carried out in Milan-Opera. The HCV infection prevalence was calculated on the imprisoned population on the January 31 2006, the data on treatment over the following 2 years. Treatment option offered to HCV chronically infected patients was then analysed, reasons for not being treated was evaluated.

**Results:**

Of the 965 inmates, 695 were enrolled in the study, 682 (98%) were males, the median age was 43 years. There were 131 (18.8%) foreigners and 564 (81.2%) Italians. HCV seroprevalence was 22.4%(95% CI:19.4%-25.7%), 60 subjects (38.4%) being HIV co-infected too. Prevalence of HCV infection was significantly higher in HIVAb positive (89.6%; 95% CI:79.7%-95.7%) than in HIVAb negative (15.15%; 95% CI 12.6%-18.3%) (p<0.001). Among Italian inmates HCVAb positivity was significantly higher than among foreigners (p=0.0154). Among HCVAb positive patients, 135 subjects were HCV-RNA positive. Forty-seven (36%) had major clinical contraindication to treatment, 18 (13%) refused the treatment, 7 (5%) moved to other Institute and 27 (20%) were not evaluated by infectious disease specialists. Fifteen patients (43%) who received treatment were considered responders, 9 (26%) were non responders/relapsers, 6 (17%) interrupted treatment due to side effects and 5 (14%) were released during treatment and lost in follow-up.

**Conclusions:**

This study indicates that the proportion of patients in a prison setting receiving diagnosis and treatment for HCV infection remained low.

## Background

HCV infection in prison inmates is widespread, surveys in correctional system show an HCV sero-prevalence ranging from 16% to 42% in U.S.A. [1], 30% to 50% in European Countries [2] and 31% to 38% among Italian inmates [3,4].

As a consequence, several national authorities published guidelines which recommend testing each incoming inmate for blood transmitted diseases. In the 16/04/2000 act issued to preserve health in correctional settings [5], the Italian Ministry of Health made it necessary not only to test and treat all intra-venous drug abuse associated infections but also to promote educational programs to improve prison inmate awareness on preventive measures and therapeutic needs, as a result HCV screening should be offered to all inmates at admission. On the other hand, in a real life setting, due to a complex array of local (in jail) and national (Italian Ministry of Justice) organization deficits, not always are inmates routinely tested for either hepatitis virus and human immunodeficiency virus (HIV). An overwhelming number of daily incoming prisoners, a shortage of adequate health facilities and trained staff, continuous recirculation of inmates among prisons located in Italy, a lack of a national health database of restricted subjects are the main reasons which hindered the carrying out of proposed acts.

Data on treatment of HCV chronic hepatitis in prisons are limited to few observational studies [6-8], small case series have been published in Italy on this specific topic [9]. In general, only a minority of patients with hepatitis C in prison can complete a full course treatment as early patient’s release or side effects or concomitant pathologies have limited the access to treatment [7,8].

The object of this study was firstly to evaluate the prevalence of HCV infection among prisoners and its epidemiological correlates, secondly to estimate the number who could be treated and HCV infected inmates who were actually treated, thirdly to calculate the number of sustained virologic responders among those treated.

## Methods

The study was designed as a single-centre cross-sectional study and was conducted among inmates of a single, large North Italian prison (Milan-Opera) where the vast majority of inmates had been sentenced for a period longer than three years. This prison was built to contain 750 inmates, but actually more than a thousand (roughly 1200) people are normally detained. It is split into male and female sections (this last section was closed in 2010) with an inner Clinical Centre with one medical ward, and an isolation area for air born infection and two AIDS-wards.

Prevalence of HCV infection was calculated on the imprisoned population on the January 31 2006, data on treatment during the following 2 years. Data collection was carried out in two steps. Firstly, all the inmates sanitary records were reviewed to asses HCV sero-status. Secondly, all the subjects either untested or with a previous negative result of more than three years underwent HCV serology. Among patients with positive HCV serology, it was evaluated the prevalence of blood HCV RNA determination. Treatment option offered to HCV chronically infected patients was then analysed, reasons for not being treated was also evaluated. Lastly, among those treated, percentages of responders and non responders to treatment were analysed.

The Ministry of Justice study approved the study and granted an official waiver to proceed as data were collected in accordance with the ethical standards, guaranteeing the anonymity of the results. All participants signed an informed consent.

### Statistical analysis

Categorical variables were described by means of count and percentage, continuous ones by means of median and range or interquartile range. Prevalence were given together with their 95% confidence interval (95% CI). Baseline patients characteristics (age, gender, nation of birth and HIV/HBV coinfection) were described for the overall sample and according to HCV infection. The Student’s *t* test was used to compare continuous variables and the Chi-square test was used to study the association between categorical variables.

Logistic regression model was fitted with HCV infection as dependent variable and nationality and age as independent variables. The statistical analysis was performed using Stata v11 (StataCorp, College Station, TX, USA).

## Results

Of the 965 inmates in Milan-Opera prison the day of surveillance, 695 were enrolled in the study (either were already tested for HCV in the previous three years (439 pts; 45.5%) or accepted to perform serological test (256 pts, 26.5%). Among the remaining 270 (28%) patient not tested, 137 resulted to be released at the time of blood test, 36 moved to another prison, 59 denied the consent to participate to the study, 1 patient deceased and 3 resulted to be not tested despite they gave consent. For the remaining 34, the reason of not being tested was unspecified. No demographic differences were observed between those tested and not tested for HCV infection (Table 1).

**Table 1:** Demographic characteristics of patients tested and not tested for HVC Ab

	**All N=965 N(%)**	**Tested for HCV infection N=695 N(%)**	**Not tested for HCV infection N=270 N(%)**	**p**
Age mean (sd)	43.59 (11.99)	43.2 (11.18)	44.58 (13.83)	0.11
Males	944(97.82)	682 (98.13)	262 (97.04)	0.33
Females	21 (2.18)	13 (1.87)	8 (2.96)	
Origin				
Africa	92(9.53)	59 (8.49)	33 (12.22)	0.23
East Europe	67(6.94)	47 (6.76)	20 (7.41)	
Italy	775(80.31)	564 (81.15)	211 (78.15)	
other	31(3.21)	25 (3.6)	6 (2.22)	

Demographic characteristics of the 695 inmates tested are summarized by Table 2. Percentages of women and foreigners are comparable with those of Italian prisoner’s population, respectively <5% and 13% [10].

**Table 2:** Demographic characteristics of patients HVC Ab posivite vs HCV Ab negative patients

	**All N=695 N(%)**	**HCV neg N=539 N(%)**	**HCV pos N=156 N(%)**	**p**	p*
Age mean (sd)	43.2 (11.18)	43.34 (11.71)	42.71 (9.14)	0.53	OR 0.24
					95% CI 0.97-1.01
					P=0.24
Origin					OR 2
Africa	59(8.49)	47 (8.72)	12 (7.69)		95% CI 1.2-3.4
East Europe	47(6.76)	44 (8.16)	3 (1.92)	0.03	p<0.01
Italy	564(81.15)	427 (79.22)	137 (87.82)		
other	25(3.6)	21 (3.9)	4 (2.56)		
Males	682 (98.13)	534 (99.07)	148 (94.87)	0.002	
Females	13 (1.87)	5 (0.93)	8 (5.13)		
HbsAg+	30(6.22)	24 (5.81)	6 (8.7)	0.42	
HIV+	67(9.64)	7 (1.3)	60 (38.46)	<0.001	

* Logistic regression model fitted with HCV infection as dependent variable and nationality and age as independent variables.

HCV seroprevalence was 22.4% (95% CI: 19.4%-25.7%) -156 subjects- with 60 subjects (38.4%) being HIV co-infected too (Table 2). Prevalence of HCV infection was significantly higher in HIVAb positive (89.6%; 95% CI: 79.7%-95.7%) than in HIVAb negative (15.15%; 95% CI 12.6%-18.3%) (p<0.001). Median CD4+ cell count in HIV/HCV confected subjects was 351 cell/mm^3^ (interquartile range: 198–492 cell/ mm^3^).

**Figure 1:**
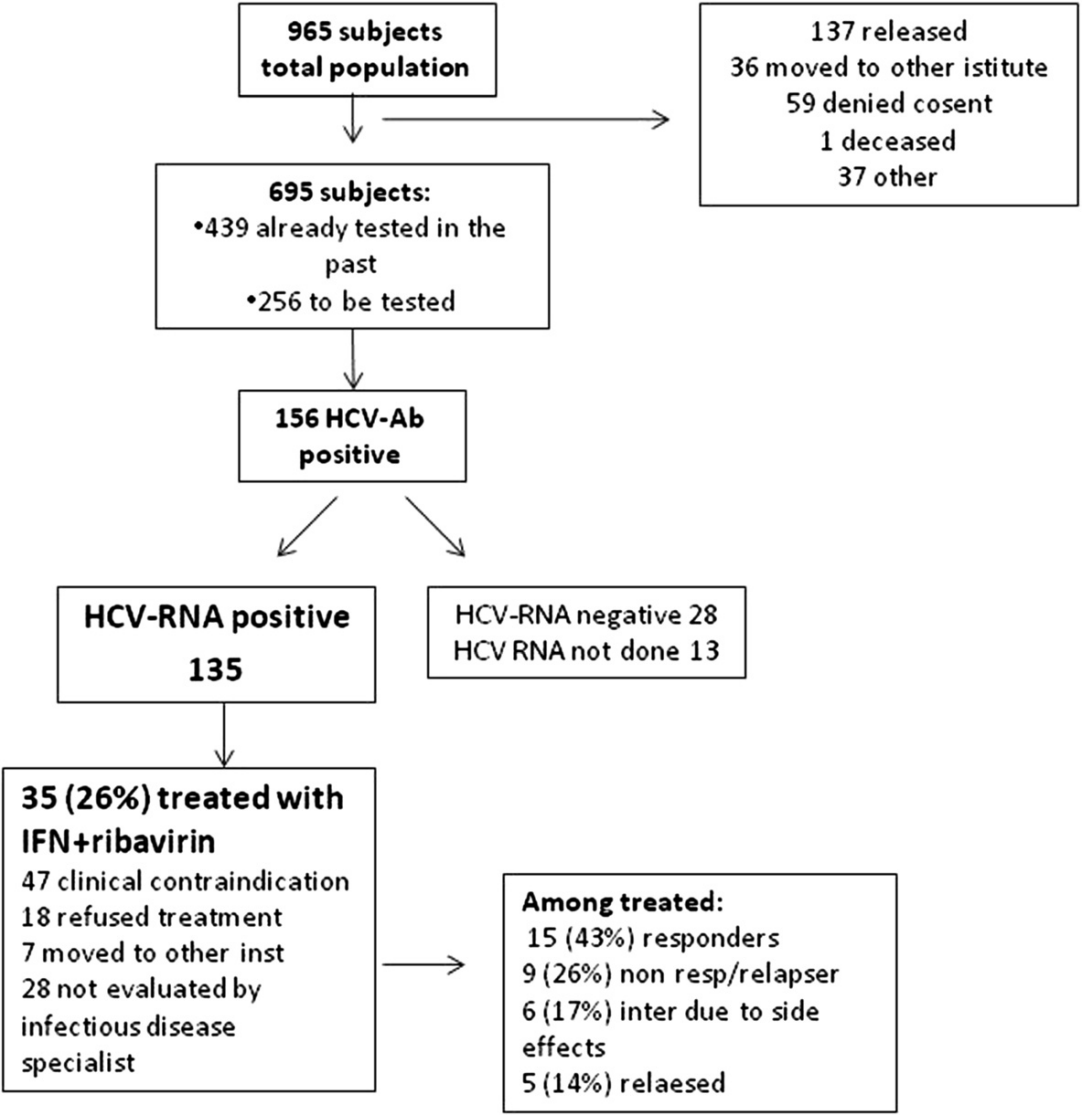
Flow diagram that summarizes the data on diagnosis and treatment of patients with HCV infection.

Out of the 564 Italian inmates, 137 (24.5%; 95% CI 20.8%-28.0%) were HCVAb positive, among foreigners 19/131 were HCVAb positive (14.5%; 95% CI: 9.0%-21.7%); namely 12 (20.3%) out of 59 African inmates, 3 (6.4%) out of 47 from East-Europe and 4 (16%) out of 25 Latino-American were HCVAb positive. Among Italian inmates HCVAb positivity was significantly higher than among foreigners (p=0.0154), this association remained statistically significant after correction for age (OR 2, 95% CI 1.2-3.4; p<0.01). In Italian inmates, HCVAb positivity was higher in age classes between 35–42 and 43–52 years (37% and 28.7%, respectively) while was lower in age classes between 18–34 and 53–82 (18 and 12%, respectively), (p<0.001). Out of the 13 women tested for HCVAb, 8 (61.5%) were positive. Among HCVAb positive patients, 135 subjects were HCV-RNA positive, 28 were HCV RNA negative and 13 did not perform any HCV RNA test (7 early release, 6 unknown) (Figure 1). Thirty-five (26%) subjects among those HCV RNA positive were evaluated by infectious disease specialist and were elected to receive treatment with IFN and ribavirin. Forty-seven (36%) had major clinical contraindication to treatment (i.e. mental illness, cirrhosis, cardiac disease, low CD4+ cell count in HIV+ subjects), 18 (13%) refused the treatment, 7 (5%) moved to other Institute and 27 (20%) were not evaluated by infectious disease specialist. Fifteen patients (43%) who received treatment were considered responders, 9 (26%) were non responders/relapsers, 6 (17%) interrupted treatment due to side effects and 5 (14%) were released during treatment and lost to follow-up.

## Discussion

The present study highlights the difficulties encountered in a screening process for HCV in a prison setting, despite dedicated personnel to this specific duty, roughly 30% of subjects did not receive the screening test, eventually only a minority of them denied the consent to participate. The problem of HCV infection in prison is extensive and troublesome, but both in US and in Europe [11,12], according to directives, correctional facilities should seriously cope with the problem of HCV infection. The CDC estimates that 12% to 35% of US inmates had chronic HCV infection [12], the majority of these patients being represented by IVDU and/or tattooing [13,14]. According to different studies, the burden of HCV infected prisoners in Italy varies between 20 and 40%, therefore considering an inmate average number of 65000 [10], roughly 10000 to 20000 patients in prison potentially need treatment (considering that roughly the 20% of patients testing antibody positive doesn’t need treatment). These data emphasize the importance of surveillance in prison [15], and counseling of patients affected from HCV infection about prevention strategies and treatment options [7,8].

The difficulties associated with HCV screening procedures in a prison setting as Opera is, are principally related to severe limitation due to a complex array of problems. First, institutional regulations and organization which cause the mobility of prisoners, who are moved from prison to prison for different reasons such as trial in cities far from the residence prison. Second, logistic problems (i.e. all the blood examination are performed outside the prison, and require time-expensive procedures to be activated) which translate into long time span between the HCV test prescription and its execution. As a consequence, despite it should be mandatory to test for hepatitis viruses and HIV all inmates, these tests are not routinely performed on admission, or, even when performed, their results can be lost during prisoner’s relocation.

Compared to Italian, foreign prisoners showed a lower prevalence of HCV infection, with African born patients, among foreigners, demonstrating the highest one, as published elsewhere [2]. Not surprisingly, women had a higher prevalence of HCV infection than men, as higher rates of Hepatitis B and C, HIV, and sexually transmitted infection, estimated at 2–10 times that of the general population were observed among imprisoned women, moreover, women had a significantly higher prevalence of all medical and psychiatric conditions and drug dependence when compared with imprisoned men [16].

In our study even if only 18% of total HCV-RNA positive inmates refused the treatment, 35 subjects (26%) eventually were treated. Twenty per cent were untreated as they did not had specialist consultation and/or did not complete diagnostic procedures mainly due to organizing deficit and logistic problems. An interplay between medical and security staff should be implemented, in order to better organize the management of health needs of patients. Even if on 35 patients only, a 43% of SVR was observed, value that is comparable to data of SVR reported in the literature [17], confirming that in prison HCV treatment is an important option to be offered to inmates. HCV prognosis is worse if not appropriately treated, as evidenced by reduced mortality in patients successfully treated [18]. In a recent study conducted in Texas, end stage liver disease mortality in prison population is approximately three times higher than that of the general population, reflecting elevated rates of HCV and HIV/HCV co-infection among prisoners [19]. Finally 14% of our treated subjects, released during treatment, were lost to follow up. As releasing from jail in Italy most of times take place with no forewarning, our patients were released without any therapeutic record and no practical possibility to continue treatment out of jail. We strongly suggest Italian correctional system to improve guidelines to release inmates with sanitary records so that they can continue any treatment once free.

The highest prevalence of HCV infection was observed among HIV positive patients. HIV-HCV-coinfected patients have higher levels of serum HCV RNA [20], an accelerated progression to end stage liver disease and a more rapid development of liver fibrosis and cirrhosis compared to those infected with HCV alone [21], moreover, when treated with antiretroviral treatment for HIV, they may show a decreased immune recovery [22]. Given the complex interplay of the two virus, HIV and HCV, it should be mandatory that HIV/HCV confected patients receive appropriate treatment for either HIV and HCV as soon as they need it. To further complicate HCV treatment in prison setting, an association between psychiatric disorders and hepatitis virus prevalence among inmates has been described [23], as result of intravenous drug abuse. Report of patients with chronic hepatitis C and psychiatric diseases treated in prison with interferon and ribavirin suggest that the treatment is possible and can be associated with discrete rate of virologic success [6,7,9,24]. An integrated approach with psychiatric support, has been described to favorably overcome the problem of psychiatric disorders in correctional settings [6] and should be advocated for all HCV patients with psychiatric co-morbidities.

The principal limitation of this study is represented by its being a single center study, so that the extent of generalizability of our findings to the prisoner’s population thus remains unclear, despite the fact the Opera represents one of the larger Italian prisons. Data on HCV prevalence reported by other Italian authors are slightly higher than the ones reported in the present study [3,4]. The reason for this difference may be explained by the fact that the population detained in Milano Opera is different from those of smaller prisons, as in Milano Opera are detained those prisoner sentenced for a period longer than three years collected from all the Country for different major crimes (that are not strictly associated with illicit drug abuse) and in smaller prisons are detained prisoners for minor offences (usually intravenous drug abusers). Moreover, despite roughly 30% of patients restricted at the time of survey were not test for HCV infection, demographic differences between those tested and not tested were not observed, not altering the reproducibility of the data. Other studies [3,4] report data on a subset of the examined population (less than 50%) and this might have biased the reproducibility of the results. Another limitation is the absence of adequate follow up as a longer observation period, including also released patients, would have been useful to evaluate the response rate to treatment.

## Conclusion

The present and other studies evidence that the proportion of patient in a prison setting receiving diagnosis and treatment for HCV infection remain low. The weight of HCV infection among HIV positive patients is worrisome, as they often can not be treated due severe immunosuppression and share the worse prognosis. An integrated health care taskforce should be encouraged and reinforced, as management of treatment of chronic hepatitis C requires the participation of specialists (hepatologist, infectious disease specialist, radiologist, psychiatrists) as well as primary care doctors and nurses.

## Abbreviations

HCV: Hepatitis C virus; CDC: Center for disease control; IVDU: Intra venus drug abuser; HIV: Human immunodeficiency virus; SVR: Sustained virologic response; Ab: Antiboby.

## Competing interests

MB received an unconditional grant from Shering-Plough for this research. The authors declare that they have no competing interests.

## Authors’ contributions

MB, SN and ES conceived of the study, participated in study design, enrolled and treated patients and drafted the manuscript. AD and CT performed the statistical analysis and reviewed the manuscript. SFA P and RR treated patients and revised the manuscript. All authors read and approved the final manuscript.

## Pre-publication history

The pre-publication history for this paper can be accessed here:

http://www.biomedcentral.com/1471-2458/13/981/prepub
